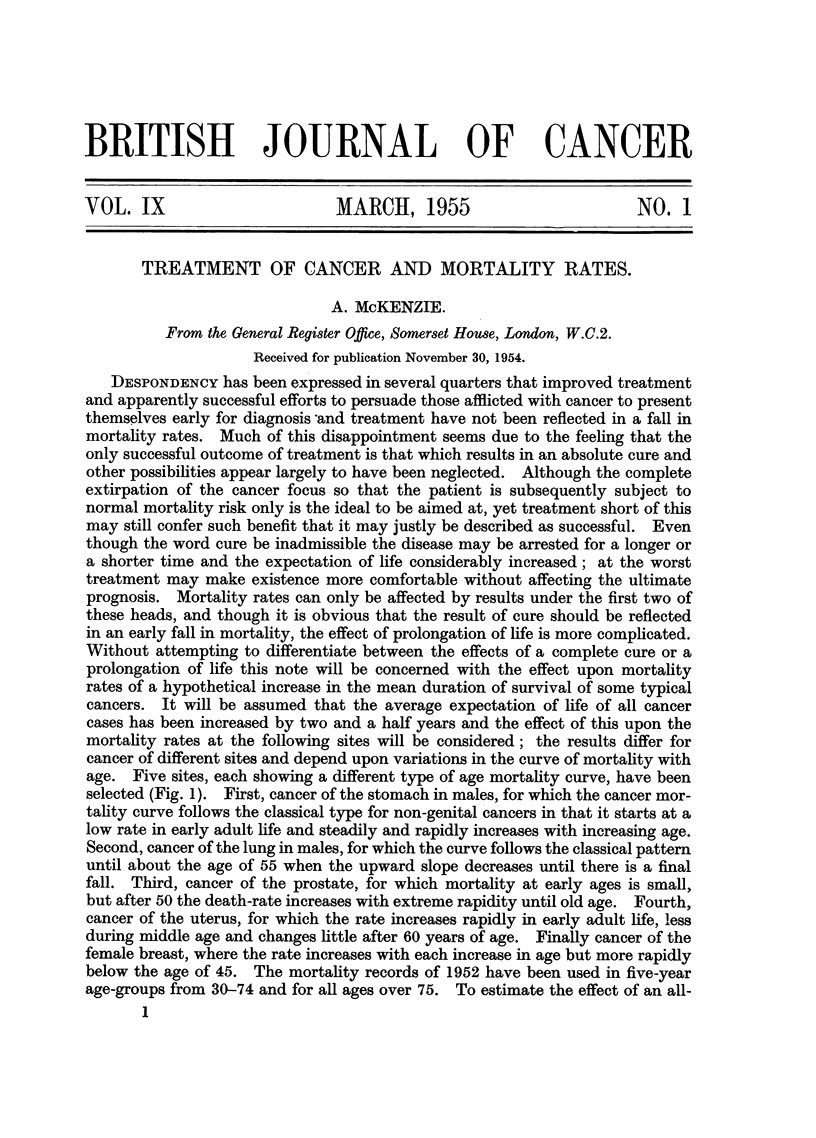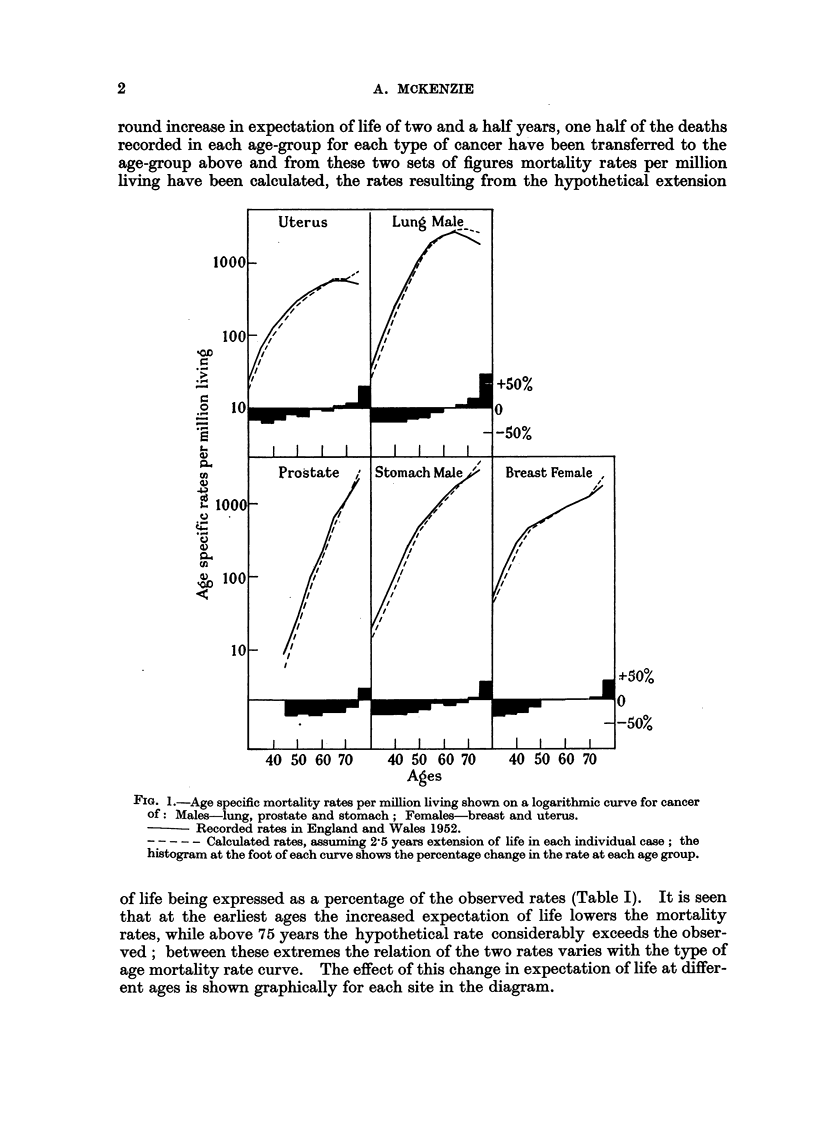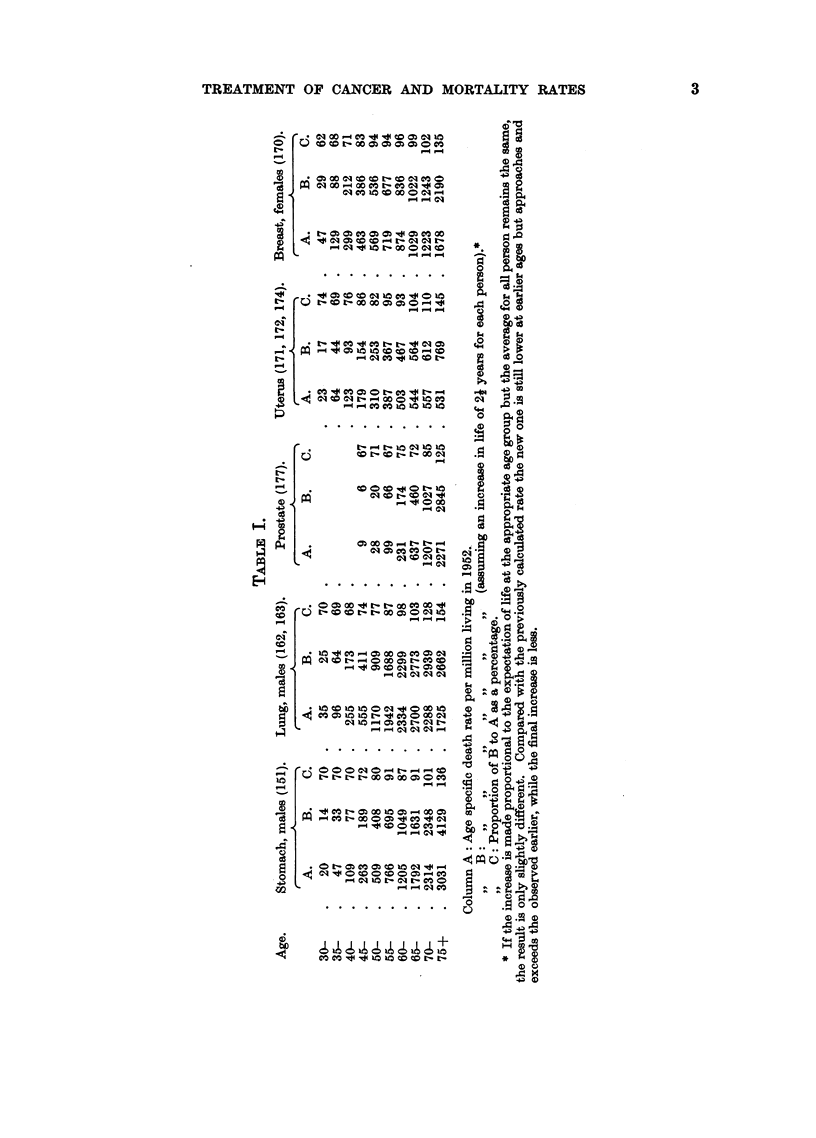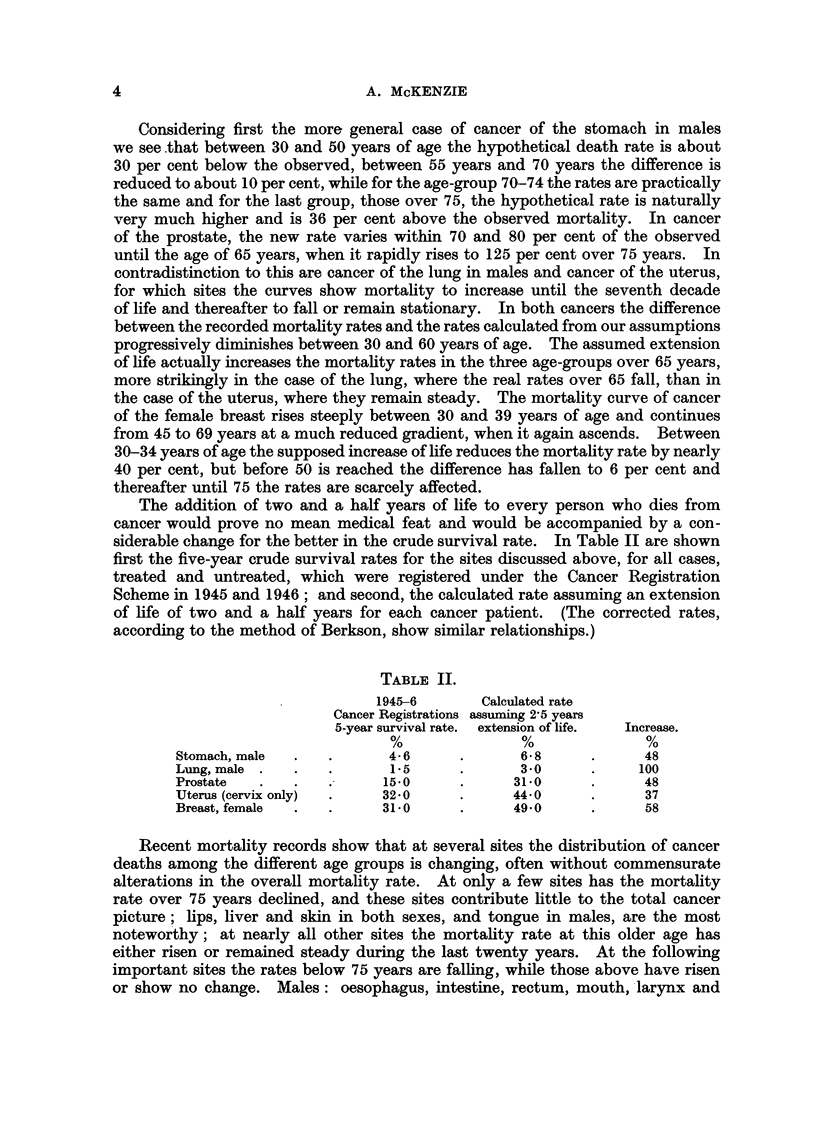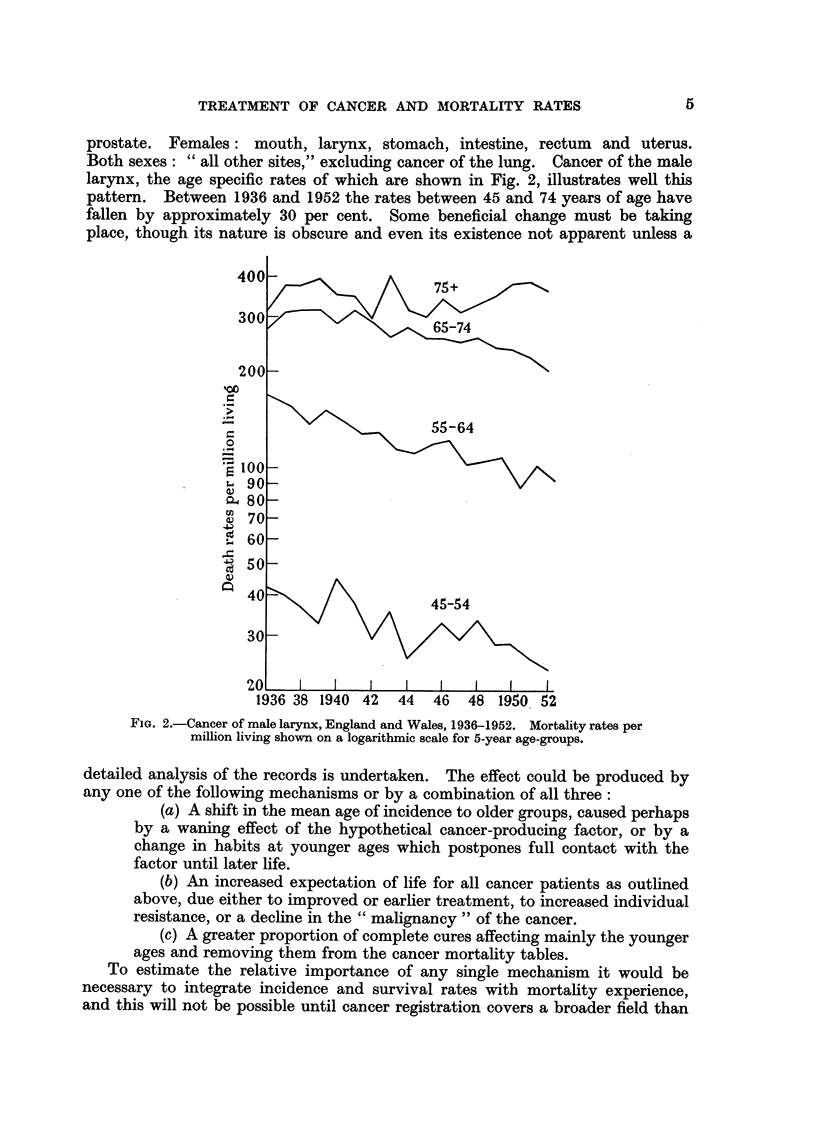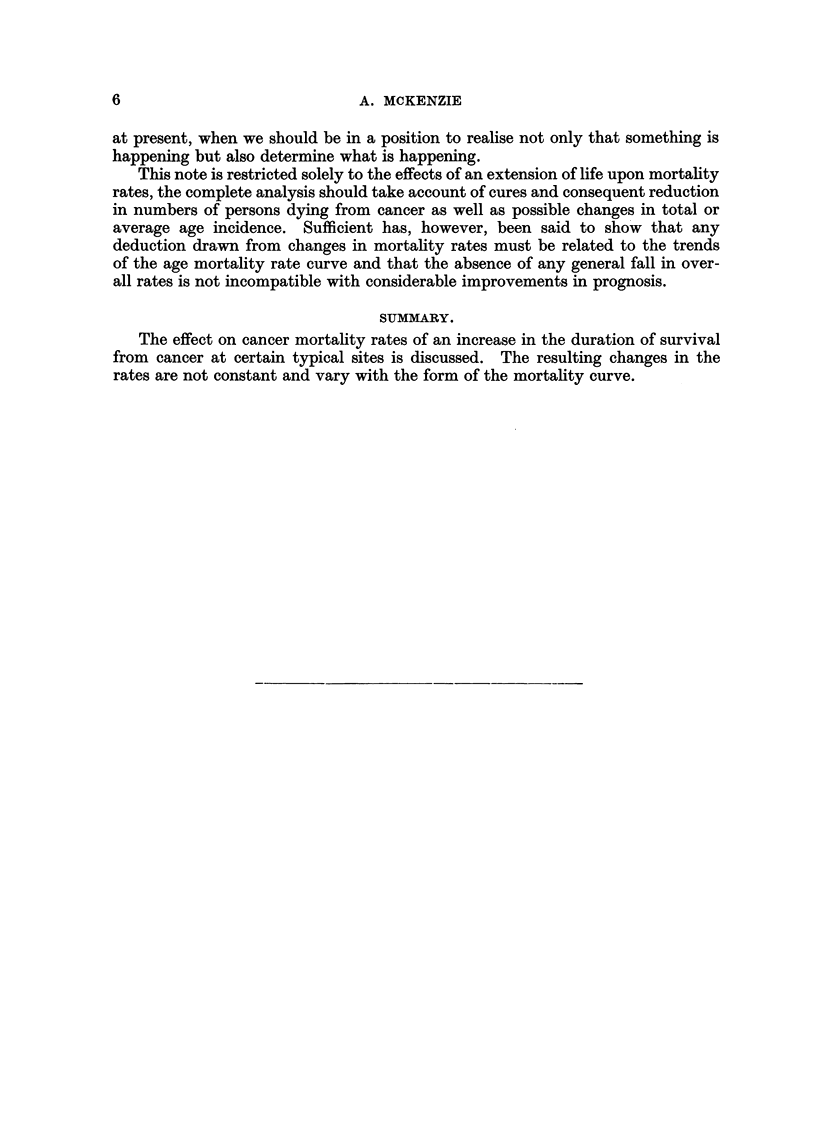# Treatment of Cancer and Mortality Rates

**DOI:** 10.1038/bjc.1955.1

**Published:** 1955-03

**Authors:** A. McKenzie


### BRITISH JOURNAL OF CANCER

VOL. IX MARCH, 1955 NO. 1

### TREATMENT OF CANCER AND MORTALITY RATES.

A. McKENZIE.

*From the General Register Office, Somerset House, London, W.C.2.*

Received for publication November 30, 1954.

DESPONDENCY has been expressed in several quarters that improved treatment and apparently successful efforts to persuade those afflicted with cancer to present themselves early for diagnosis and treatment have not been reflected in a fall in mortality rates. Much of this disappointment seems due to the feeling that the only successful outcome of treatment is that which results in an absolute cure and other possibilities appear largely to have been neglected. Although the complete extirpation of the cancer focus so that the patient is subsequently subject to normal mortality risk only is the ideal to be aimed at, yet treatment short of this may still confer such benefit that it may justly be described as successful. Even though the word cure be inadmissible the disease may be arrested for a longer or a shorter time and the expectation of life considerably increased ; at the worst treatment may make existence more comfortable without affecting the ultimate prognosis. Mortality rates can only be affected by results under the first two of these heads, and though it is obvious that the result of cure should be reflected in an early fall in mortality, the effect of prolongation of life is more complicated. Without attempting to differentiate between the effects of a complete cure or a prolongation of life this note will be concerned with the effect upon mortality rates of a hypothetical increase in the mean duration of survival of some typical cancers. It will be assumed that the average expectation of life of all cancer cases has been increased by two and a half years and the effect of this upon the mortality rates at the following sites will be considered ; the results differ for cancer of different sites and depend upon variations in the curve of mortality with age. Five sites, each showing a different type of age mortality curve, have been selected (Fig. 1). First, cancer of the stomach in males, for which the cancer mortality curve follows the classical type for non-genital cancers in that it starts at a low rate in early adult life and steadily and rapidly increases with increasing age. Second, cancer of the lung in males, for which the curve follows the classical pattern until about the age of 55 when the upward slope decreases until there is a final fall. Third, cancer of the prostate, for which mortality at early ages is small, but after 50 the death-rate increases with extreme rapidity until old age. Fourth, cancer of the uterus, for which the rate increases rapidly in early adult life, less during middle age and changes little after 60 years of age. Finally cancer of the female breast, where the rate increases with each increase in age but more rapidly below the age of 45. The mortality records of 1952 have been used in five-year age-groups from 30–74 and for all ages over 75. To estimate the effect of an allround increase in expectation of life of two and a half years, one half of the deaths recorded in each age-group for each type of cancer have been transferred to the age-group above and from these two sets of figures mortality rates per million living have been calculated, the rates resulting from the hypothetical extension

FIG. 1.—Age specific mortality rates per million living shown on a logarithmic curve for cancer of: Males—lung, prostate and stomach; Females—breast and uterus.
——— Recorded rates in England and Wales 1952.
– – – – – Calculated rates, assuming 2·5 years extension of life in each individual case; the histogram at the foot of each curve shows the percentage change in the rate at each age group.

of life being expressed as a percentage of the observed rates (Table I). It is seen that at the earliest ages the increased expectation of life lowers the mortality rates, while above 75 years the hypothetical rate considerably exceeds the observed; between these extremes the relation of the two rates varies with the type of age mortality rate curve. The effect of this change in expectation of life at different ages is shown graphically for each site in the diagram.

### TABLE I.

Considering first the more general case of cancer of the stomach in males we see that between 30 and 50 years of age the hypothetical death rate is about 30 per cent below the observed, between 55 years and 70 years the difference is reduced to about 10 per cent, while for the age-group 70–74 the rates are practically the same and for the last group, those over 75, the hypothetical rate is naturally very much higher and is 36 per cent above the observed mortality. In cancer of the prostate, the new rate varies within 70 and 80 per cent of the observed until the age of 65 years, when it rapidly rises to 125 per cent over 75 years. In contradistinction to this are cancer of the lung in males and cancer of the uterus, for which sites the curves show mortality to increase until the seventh decade of life and thereafter to fall or remain stationary. In both cancers the difference between the recorded mortality rates and the rates calculated from our assumptions progressively diminishes between 30 and 60 years of age. The assumed extension of life actually increases the mortality rates in the three age-groups over 65 years, more strikingly in the case of the lung, where the real rates over 65 fall, than in the case of the uterus, where they remain steady. The mortality curve of cancer of the female breast rises steeply between 30 and 39 years of age and continues from 45 to 69 years at a much reduced gradient, when it again ascends. Between 30–34 years of age the supposed increase of life reduces the mortality rate by nearly 40 per cent, but before 50 is reached the difference has fallen to 6 per cent and thereafter until 75 the rates are scarcely affected.

The addition of two and a half years of life to every person who dies from cancer would prove no mean medical feat and would be accompanied by a considerable change for the better in the crude survival rate. In Table II are shown first the five-year crude survival rates for the sites discussed above, for all cases, treated and untreated, which were registered under the Cancer Registration Scheme in 1945 and 1946 ; and second, the calculated rate assuming an extension of life of two and a half years for each cancer patient. (The corrected rates, according to the method of Berkson, show similar relationships.)

TABLE II.

| | 1945–6 Cancer Registrations 5-year survival rate. | Calculated rate assuming 2·5 years extension of life. | Increase. |
|---|---|---|---|
| | % | % | % |
| Stomach, male | 4·6 | 6·8 | 48 |
| Lung, male | 1·5 | 3·0 | 100 |
| Prostate | 15·0 | 31·0 | 48 |
| Uterus (cervix only) | 32·0 | 44·0 | 37 |
| Breast, female | 31·0 | 49·0 | 58 |

Recent mortality records show that at several sites the distribution of cancer deaths among the different age groups is changing, often without commensurate alterations in the overall mortality rate. At only a few sites has the mortality rate over 75 years declined, and these sites contribute little to the total cancer picture ; lips, liver and skin in both sexes, and tongue in males, are the most noteworthy ; at nearly all other sites the mortality rate at this older age has either risen or remained steady during the last twenty years. At the following important sites the rates below 75 years are falling, while those above have risen or show no change. Males : oesophagus, intestine, rectum, mouth, larynx and prostate. Females: mouth, larynx, stomach, intestine, rectum and uterus. Both sexes: "all other sites," excluding cancer of the lung. Cancer of the male larynx, the age specific rates of which are shown in Fig. 2, illustrates well this pattern. Between 1936 and 1952 the rates between 45 and 74 years of age have fallen by approximately 30 per cent. Some beneficial change must be taking place, though its nature is obscure and even its existence not apparent unless a

FIG. 2.—Cancer of male larynx, England and Wales, 1936–1952. Mortality rates per million living shown on a logarithmic scale for 5-year age-groups.

detailed analysis of the records is undertaken. The effect could be produced by any one of the following mechanisms or by a combination of all three:

(*a*) A shift in the mean age of incidence to older groups, caused perhaps by a waning effect of the hypothetical cancer-producing factor, or by a change in habits at younger ages which postpones full contact with the factor until later life.

(*b*) An increased expectation of life for all cancer patients as outlined above, due either to improved or earlier treatment, to increased individual resistance, or a decline in the "malignancy" of the cancer.

(*c*) A greater proportion of complete cures affecting mainly the younger ages and removing them from the cancer mortality tables.

To estimate the relative importance of any single mechanism it would be necessary to integrate incidence and survival rates with mortality experience, and this will not be possible until cancer registration covers a broader field than at present, when we should be in a position to realise not only that something is happening but also determine what is happening.

This note is restricted solely to the effects of an extension of life upon mortality rates, the complete analysis should take account of cures and consequent reduction in numbers of persons dying from cancer as well as possible changes in total or average age incidence. Sufficient has, however, been said to show that any deduction drawn from changes in mortality rates must be related to the trends of the age mortality rate curve and that the absence of any general fall in overall rates is not incompatible with considerable improvements in prognosis.

### SUMMARY.

The effect on cancer mortality rates of an increase in the duration of survival from cancer at certain typical sites is discussed. The resulting changes in the rates are not constant and vary with the form of the mortality curve.